# Performance of Multi-Gradient Biomimetic POSS-Toughened CFRP Laminates with a Pearl-Inspired Hard-Soft Interfacial Architecture

**DOI:** 10.3390/polym18182199

**Published:** 2026-09-09

**Authors:** Gefeng Xiang, Shuwei Sun, Yujie Wang, Baorui Jia, Changqing Li, Mingli Qin

**Affiliations:** 1 Institute for Advanced Materials and Technology, University of Science and Technology Beijing, Beijing 100083, China; m202420555@xs.ustb.edu.cn (G.X.); jiabaorui@ustb.edu.cn (B.J.); 2 Scientific and Technological Innovation Center, Beijing 100012, China; ssw9604@163.com (S.S.); 15122435509@126.com (Y.W.)

**Keywords:** carbon-fiber-reinforced polymer, polyhedral oligomeric silsesquioxane, pearl-inspired gradient interface, compression after impact

## Abstract

Carbon-fiber-reinforced epoxy (CFRP) composites are widely used in aerospace structures because of their high specific strength and modulus; however, matrix cracking and interlaminar delamination induced by low-velocity impact compromise their structural safety. To overcome the difficulty of simultaneously improving interfacial strength and impact resistance through uniform nanomodification, a pearl-inspired gradient interfacial architecture incorporating a through-thickness distribution of polyhedral oligomeric silsesquioxane (POSS) was developed and compared with unmodified and uniformly modified CFRP laminates. SEM/EDS, TEM, double-cantilever-beam testing, low-velocity impact testing, compression-after-impact (CAI) testing, and micro-CT were combined to examine the effects of POSS spatial distribution on interfacial morphology, Mode I interlaminar fracture toughness, and impact-damage evolution. POSS incorporation increased interfacial roughness and produced nanoscale POSS-rich domains within the epoxy matrix. Although uniform addition of 0.5 wt% POSS increased the mean CAI strength to 142 MPa, the Mode I interlaminar fracture toughness decreased to 0.479 kJ·m^−2^, revealing a trade-off in performance. By contrast, the gradient interface combined a high-POSS region with low-POSS regions that retained greater matrix continuity, thereby integrating material modification with through-thickness structural regulation. The gradient laminate showed more segmented crack paths and damage dispersion while suppressing continuous delamination. In the present tests, the gradient CFRP laminate exhibited a Mode I interlaminar fracture toughness of 1.342 kJ·m^−2^ (*n* = 1) and a mean CAI strength of 192 MPa (*n* = 3), corresponding to descriptive increases of approximately 148% and 113.3%, respectively, relative to the unmodified laminate. These results indicate that the response of the tested laminates depends not only on POSS content but also on its spatial distribution, providing an interfacial-design strategy for high-damage-tolerance CFRP laminates.

## 1. Introduction

Carbon-fiber-reinforced polymer (CFRP) composites based on epoxy matrices combine high specific strength, high specific stiffness, low density, corrosion resistance, and structural design flexibility, and are therefore widely used in aerospace, rail transportation, automotive, and other high-performance structures [1,2,3]. Unlike metals, the load-bearing performance of CFRP composites depends strongly on the fiber lay-up, matrix toughness, and fiber/matrix interfacial condition [4]. During manufacturing, assembly, and service, laminates may be subjected to low-velocity impacts caused by dropped tools, debris, or foreign objects. Such impacts often produce barely visible surface damage but substantial internal matrix cracking and delamination. Under subsequent compressive loading, the damaged region may undergo local bending or buckling and further crack growth, resulting in a marked reduction in compression-after-impact (CAI) strength [5,6,7]. Suppressing impact-induced delamination and crack propagation while preserving residual load-bearing capacity is therefore a central challenge in the damage-tolerant design of CFRP laminates.

Current approaches to this problem include matrix toughening, nanofiller modification, interlaminar toughening, fiber/matrix interface reinforcement, and structural hybridization [4,7,8,9,10,11,12,13,14,15,16,17,18,19]. Rubber particles, thermoplastic constituents, and core–shell rubber particles can enhance the plastic deformability of epoxy matrices and reduce indentation depth and damage area after impact. Carbon nanotubes (CNTs), carbon nanofibers (CNFs), graphene, and their derivatives can improve interlaminar fracture toughness and impact-damage resistance through crack bridging, crack deflection, fiber pull-out, and interfacial friction [7,9,10,15,16,17]. Ravindran et al. [9] improved the delamination resistance and impact-damage tolerance of CFRP composites through multiscale fiber toughening. Lai et al. [7] used a magnetic field to align CNTs through the thickness, forming nanoscale bridges in the interlaminar region and thereby improving interlaminar fracture toughness and impact resistance. These studies demonstrate that the spatial location of a toughening phase and its interaction with propagating cracks can be as important as its intrinsic material properties.

Polyhedral oligomeric silsesquioxane (POSS) possesses a nanoscale inorganic Si-O-Si cage and tailorable organic functional groups, thereby combining inorganic structural stability with organic compatibility and making it suitable for epoxy toughening and carbon-fiber interface modification [12,18,19,20,21,22]. Mahfuz et al. [20] improved fiber/matrix interfacial integrity by coating carbon fibers with POSS, which enhanced the mechanical performance and environmental durability of the composites. Liu et al. [12] further demonstrated that a nanoemulsion-encapsulated POSS sizing agent improves interfacial wettability, hygrothermal stability, and the mechanical properties of CFRP composites. These findings establish POSS as an effective interfacial modifier and interlaminar toughening agent. Nevertheless, most studies have focused on POSS type, content, sizing route, and uniform dispersion, whereas its through-thickness distribution and relationship with impact-damage evolution remain insufficiently understood. In addition, the reinforcing and toughening benefits of POSS exhibit a concentration-dependent limit; consequently, uniform modification at a single POSS content reaches a performance bottleneck [23,24].

Damage caused by a low-velocity impact is not uniformly distributed through the laminate thickness. The impact-side region is dominated by contact compression, indentation, and matrix damage; the middle layers are susceptible to shear-driven delamination; and the back face often develops matrix cracks and fiber damage under flexural tension [4,25,26,27]. Uniform modification throughout the laminate therefore cannot readily satisfy the distinct requirements for surface indentation resistance, internal crack arrest, and back-face deformation accommodation. Natural nacre combines hard layers and flexible interfaces, laid with multiple layered interfaces, offering high strength and impact resistance. Its exceptional toughness does not arise from a single high-strength constituent, but from hard–soft synergy, crack deflection, interfacial friction, and multiscale energy dissipation [28,29,30,31]. Biomimetic laminates, staggered architectures, hybrid lay-ups, and functionally graded designs have consequently been explored for impact-resistant composites [26,32,33,34,35,36], indicating that through-thickness structural design is an effective means of regulating crack paths and improving damage tolerance. Recent continuous-fiber additive-manufacturing studies further demonstrate increasing freedom to tailor reinforcement paths, material placement, and interfacial topology [37,38,39]. In the present work, the pearl/nacre analogy is functional rather than a direct reproduction of nacre’s brick-and-mortar microstructure: the design adopts layered heterogeneity and spatially varying interfacial states to regulate crack propagation and deformation accommodation.

To address the deterioration of individual properties associated with uniform POSS modification at a single concentration and to further improve the strength and impact resistance of CFRP laminates, a multi-gradient POSS-toughened CFRP laminate with a pearl-inspired gradient interfacial architecture was developed. Three epoxy systems containing different POSS concentrations were applied to separate through-thickness regions to form a three-level gradient interface. This design avoids the structural uniformity of single-concentration modification while retaining the same lay-up equipment and curing cycle, although preparing and applying three resin formulations adds handling and process-control effort. The gradient architecture substantially improved both strength and impact resistance: the Mode I interlaminar fracture toughness reached 1.342 kJ·m^−2^, while the mean CAI strength reached 192 MPa, corresponding to descriptive increases of approximately 148% and 113.3%, respectively, relative to the unmodified laminate. In addition to demonstrating the performance advantages of the gradient architecture, this study clarifies the relationship between POSS content and individual mechanical properties and provides a quantifiable strategy for improving the impact resistance of CFRP laminates. Unlike approaches that alter fiber architecture, add distinct interleaves, or use digitally programmed reinforcement paths, this study keeps the same woven-fiber lay-up and uses the through-thickness concentration of a single interfacial modifier, POSS, as the design variable. The 0.5/0.3/0 wt% configuration is the best-performing one within the tested set, not a universal optimum.

## 2. Experimental

### 2.1. Fabrication of Gradient POSS-Toughened CFRP Laminates

Four types of CFRP laminate were fabricated: an unmodified laminate, uniformly modified laminates containing 0.3 wt% and 0.5 wt% POSS, and a gradient-POSS laminate. The reinforcement consisted of ten plies of orthogonally woven carbon-fiber fabric (Zhongfu Shenying T700 SYT49S-12K, Provided by Shandong Institute of Non-metallic Materials (Jinan, China)). A WB-30 epoxy system was used with an epoxy-to-curing-agent mass ratio of 100:28. Glycidoxypropyl cyclotetrasiloxane (POSS) was supplied by Xi’an Qiyue Technology Co., Ltd. (Xi’an, China). Synergistic modification using POSS at the carbon-fiber/epoxy interface has been shown to improve interfacial wettability, interlaminar shear strength, and fracture toughness [19,22].

**Figure 1:**
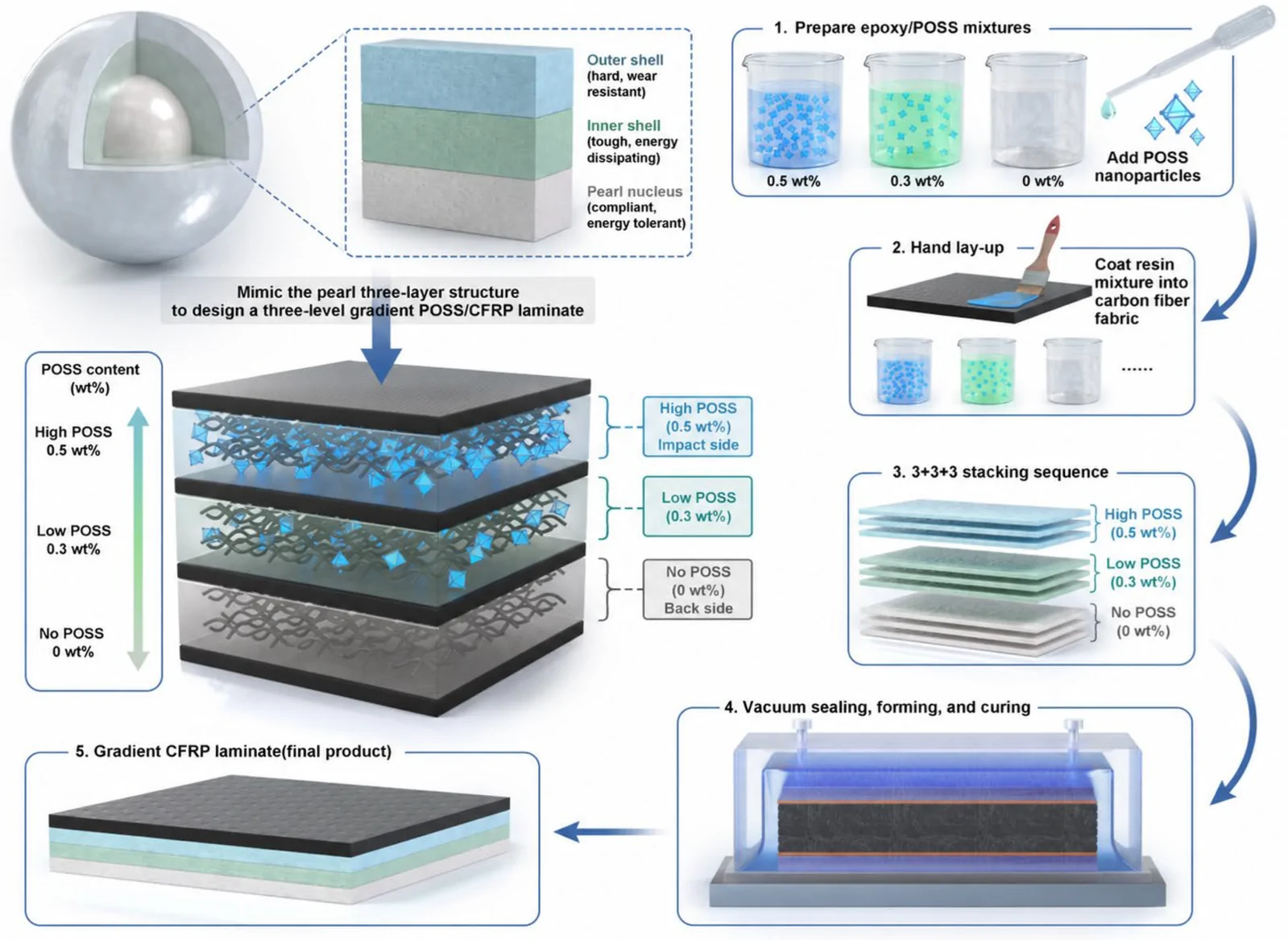
Schematic of the fabrication of the pearl-inspired gradient CFRP laminate.

As illustrated by the pearl cross-section in Figure 1, a CFRP laminate containing three through-thickness POSS concentrations was designed as a functional analogy to the layered heterogeneity of pearl/nacre rather than as a direct replication of nacre’s brick-and-mortar microstructure. The three regions contained 0.5 wt% POSS, 0.3 wt% POSS, and no POSS, respectively. A higher POSS content provides a greater improvement in impact resistance, whereas its flexural performance is slightly lower than that obtained at a lower POSS content. During impact-induced bending, the impact face sustains the highest contact load, whereas the back face experiences greater flexural tension and requires sufficient deformability. Accordingly, the 0.5 wt% POSS region was placed on the impact side, the 0.3 wt% POSS region was used as the intermediate layer, and the unmodified region was placed on the back side. The nominal POSS formulation was therefore designed to decrease stepwise from the impact face to the back face. Because the EDS mapping is semi-quantitative, it provides spatial rather than fully calibrated concentration information. The observed Si-signal differences suggest that the intended stepwise distribution remained spatially distinguishable after impregnation and curing, while some local redistribution of POSS across adjacent interfaces may have occurred during these processes.

Before fabrication, POSS was added to the liquid epoxy at the prescribed mass fraction and magnetically stirred to ensure adequate mixing before addition of the curing agent. The same resin/POSS formulation was applied to every interlayer in the unmodified, 0.3 wt%, and 0.5 wt% laminates. For the gradient laminate, the nine interlayers were divided into three through-thickness regions and impregnated with resin systems containing 0.5 wt%, 0.3 wt%, and 0 wt% POSS, respectively. The total amount of resin used across all groups of resin systems was kept consistent to reduce the impact of resin content differences on mechanical properties. The lay-up dimensions were 350 mm × 350 mm, with the warp and weft directions aligned with the specimen length and width. The laminates were vacuum sealed, held at 90 °C for 1 h, heated to 120 °C and cured for 3 h, and then cooled to room temperature. The resulting laminate thickness was approximately 3.5 mm.

### 2.2. Mode I Interlaminar Fracture Toughness and Compression-After-Impact Tests

Mode I interlaminar fracture toughness was measured using double-cantilever-beam (DCB) tests, and the test procedure and data reduction followed ASTM D5528/D5528M, including the corrected beam theory for determining GIC [40]. Piano hinges were bonded to one end of each specimen using an epoxy adhesive (Araldite 420, supplied by Huntsman^®^, Huntsman Chemical Trading (Shanghai) Co., Ltd., (Shanghai, China)). The specimens measured 200 mm × 22 mm and consisted of ten woven-fabric plies. A 10 μm polytetrafluoroethylene (PTFE) film was inserted between the fifth and sixth plies during lay-up to create a starter crack. DCB tests were conducted on a 10 kN Instron testing machine at an opening-displacement rate of 1.0 mm/min. Delamination propagated along the specimen mid-plane as the opening displacement increased. Crack growth occurred in short stick-slip increments, and the applied load was recorded for each increment. The Mode I critical strain-energy release rate, or interlaminar fracture toughness (GIC), was calculated for each crack increment using the corrected beam theory specified in ASTM D5528 [40]. The Mode I interlaminar fracture toughness was calculated using Equation (1):

(1) $$\mathrm{GIC}=\frac{3P_{c}\delta_{c}}{2b(a+|∆l|)}$$

where

P_c_ is the critical load (N);

δ_c_ is the displacement at the critical load for Mode I fracture (mm);

b is the specimen width (mm);

a is the delamination length (mm);

|Δl| is the correction factor accounting for crack-tip rotation and transverse displacement.

The initiation Mode I interlaminar fracture toughness was determined using the VIS method. The load and crack-opening displacement corresponding to the first visually detected crack extension ahead of the PTFE insert were used in the calculation.

**Figure 2:**
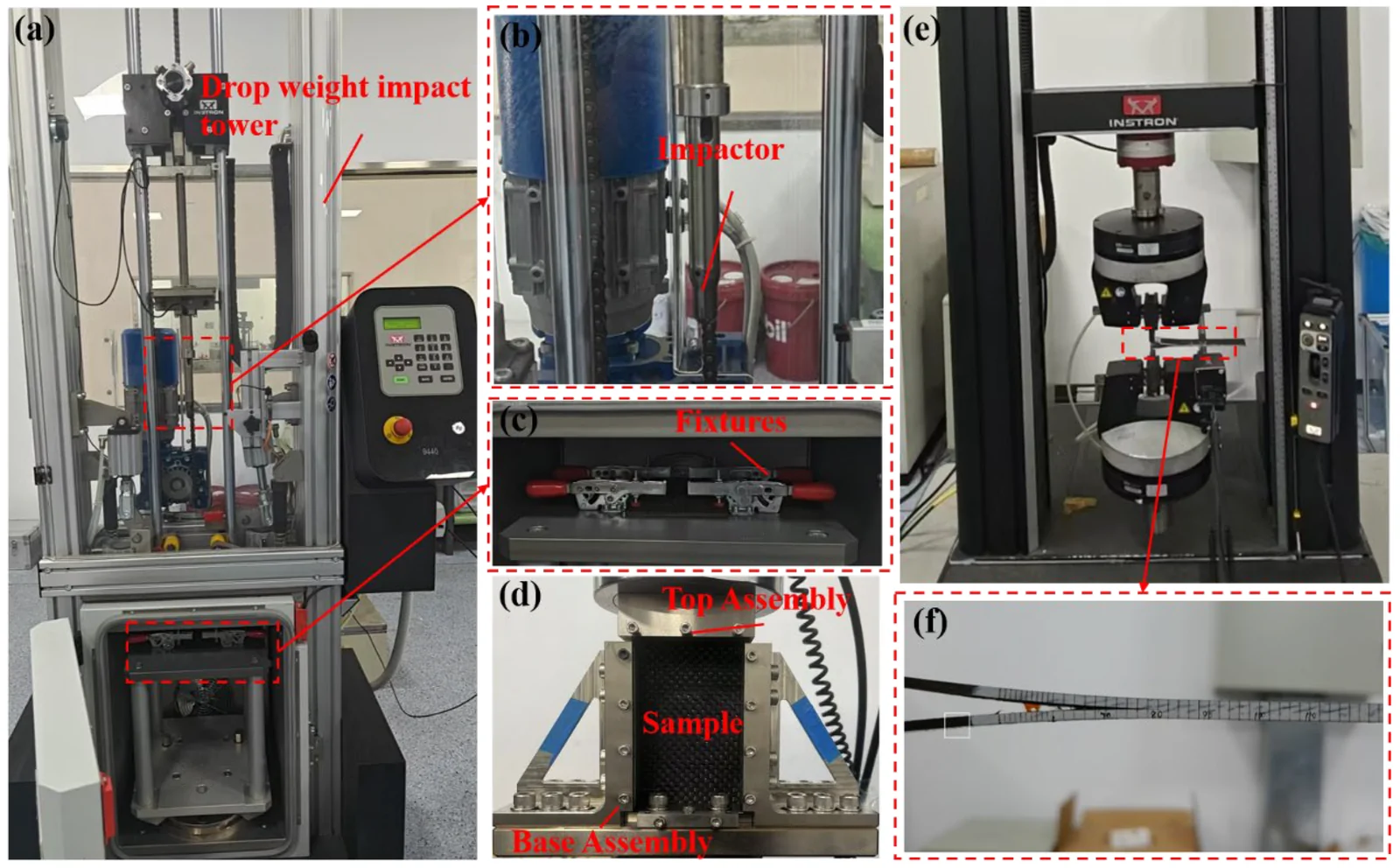
Experimental setups for drop-weight impact, compression after impact, and DCB testing: (**a**) drop-weight impact tester; (**b**) hemispherical impactor; (**c**) clamping fixture; (**d**) CAI anti-buckling fixture; (**e**) DCB test setup; (**f**) DCB specimen during testing.

Drop-weight impact tests were performed in accordance with ASTM D7136/D7136M [41] using an Instron 9440 drop-weight impact tester, as shown in Figure 2a. A hemispherical impactor was used (Figure 2b), and the specimen dimensions were 150 mm × 100 mm. The impact energy was 27.06 J, the impact velocity was 3.08 m/s, the impactor mass was 5.632 kg, and the hemispherical impactor diameter was 16 mm. Low-velocity impact can induce internal matrix cracking, interlaminar delamination, and localized fiber damage; this test was therefore used to evaluate the damage resistance of the laminates [42,43,44].

Compression-after-impact tests were conducted in accordance with ASTM D7137/D7137M [45]. Each specimen was vertically mounted in an anti-buckling fixture, as shown in Figure 2c,d, and loaded on a 250 kN universal testing machine at a displacement rate of 1.25 mm/min. The CAI strength was calculated using Equation (2) to quantify the residual load-bearing capacity of the impacted laminate [45]. For the DCB test, one specimen was tested for each laminate configuration (*n* = 1); therefore, the reported GIC values are individual measurements and no standard deviation, coefficient of variation, or statistical-significance analysis was calculated for GIC. For low-velocity impact followed by CAI testing, three specimens were tested for each laminate configuration (*n* = 3). CAI strengths are reported as mean ± standard deviation, We added error bars when plotting. Given the limited CAI sample size, the between-group differences are discussed descriptively rather than as statistically validated effects.

(2) $$\sigma_{\mathrm{CAI}}=\frac{P_{m}}{b\cdot h}$$

where

σ_CAI_ is the compression-after-impact strength (MPa);

P_m_ is the maximum applied load (N);

b is the specimen width (mm);

h is the specimen thickness (mm).

### 2.3. Microstructural Characterization and Damage Inspection

The through-thickness cross-sectional morphology and fiber/matrix interfaces were examined using a field-emission scanning electron microscope (SEM; Hitachi SU8600, Tokyo, Japan). An energy-dispersive X-ray spectroscopy system (EDS; Oxford Xplore-65, High Wycombe, UK) was used to map C, O, and Si and thereby assess the distribution of POSS in the interlaminar regions and its association with the CFRP microstructure. CFRP specimens were sectioned using a diamond-wire saw. Large-area through-thickness EDS maps were also acquired for all four laminate configurations to assess spatial changes in the Si signal.

The nanoscale through-thickness interfacial morphologies of the unmodified and gradient-POSS CFRP laminates were examined using transmission electron microscopy (TEM; FEI Talos F200i, Waltham, MA, USA). Interfacial lamellae were extracted in situ using a DB500 dual-beam focused-ion-beam scanning electron microscope (Guoyi, Hefei, China) and were progressively thinned and cleaned with a low-energy ion beam. Bright-field TEM imaging, EDX elemental mapping, and selected-area electron diffraction (SAED) were subsequently performed.

Internal crack morphologies after impact were examined nondestructively using X-ray micro-computed tomography (micro-CT; YXLON FF35CT, Hamburg, Germany). The scanning parameters were 140 μA and 100 kV, with a rotation increment of 0.2°, an exposure time of 333 ms per projection, and a voxel size of 34.75 μm. The reconstructed volumes were post-processed in VG Studio Max to extract the damaged regions, crack morphologies, and delamination characteristics.

## 3. Results and Discussion

### 3.1. SEM and TEM of Interfacial Morphology

**Figure 3:**
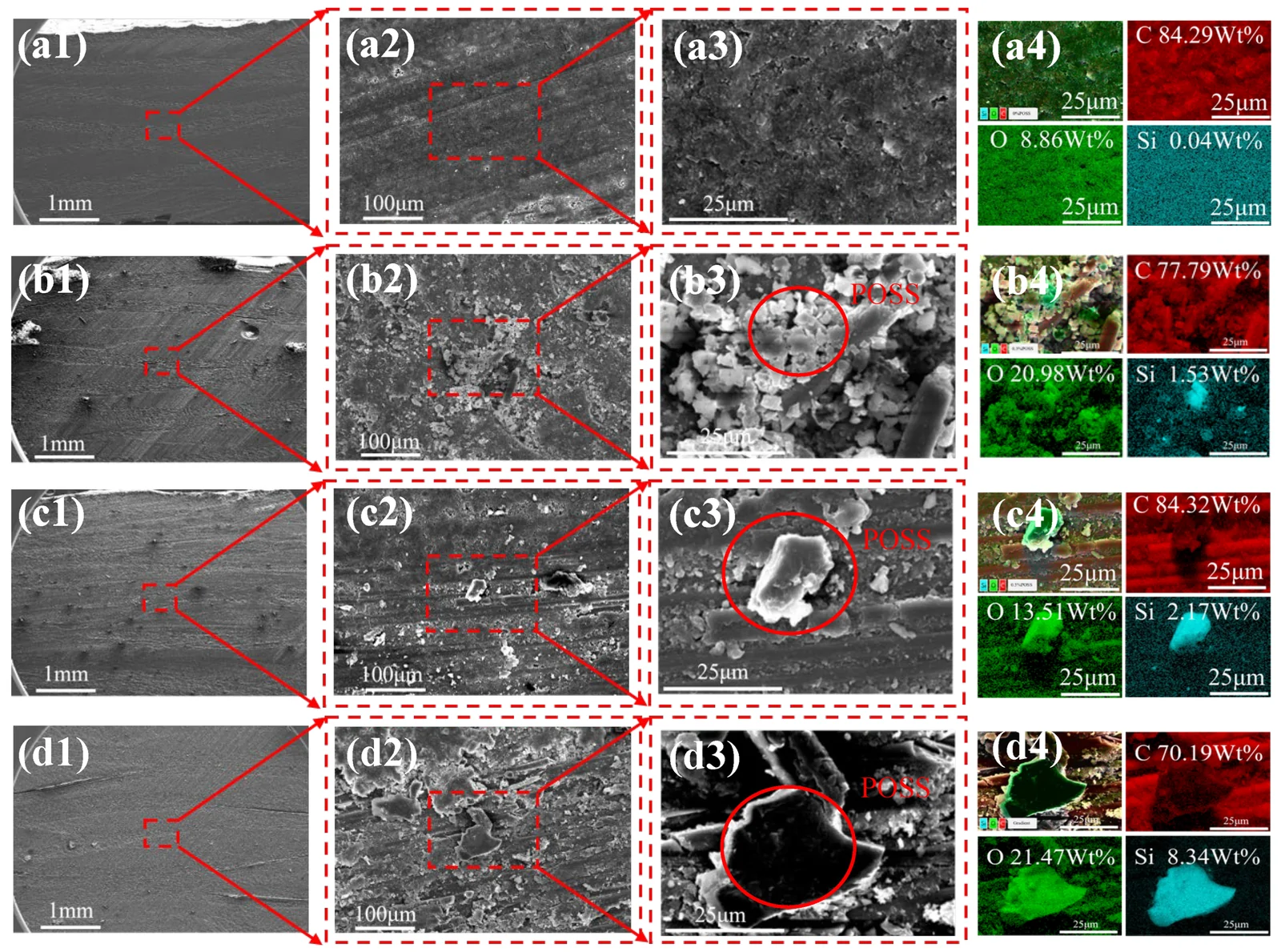
SEM images and EDS elemental maps of CFRP laminates with different POSS contents: (**a1**–**a4**) 0 wt% POSS; (**b1**–**b4**) 0.3 wt% POSS; (**c1**–**c4**) 0.5 wt% POSS; (**d1**–**d4**) gradient POSS.

Figure 3 presents SEM images and EDS elemental maps of the through-thickness cross-sections of CFRP laminates containing different POSS contents. The unmodified laminate exhibited a relatively flat fracture surface dominated locally by a continuous resin phase, and its EDS spectrum showed only a weak Si signal. The Si content in Figure 3(a4) was below 0.1 wt%, consistent with the absence of intentionally added POSS. The EDS results showed an overall increase in the local Si content in the 0.3 wt%, 0.5 wt%, and gradient groups, and the Si-enriched regions generally coincided with the high-contrast particles observed in the SEM images. After POSS incorporation, particulate or block-like high-contrast regions appeared in the through-thickness cross-sections, and the surface roughness increased with increasing POSS content. The unmodified interface was comparatively smooth and showed little mechanical interlocking between the carbon fibers and epoxy matrix. Under impact loading, such a smooth interface can form a low-resistance crack path along which cracks propagate rapidly, promoting interfacial debonding, interlaminar cracking, and fiber pull-out [46]. In the uniformly modified laminates, the 0.3 wt% group contained relatively well-dispersed particles with small local agglomerates, whereas the 0.5 wt% group exhibited larger enrichment domains and shorter interparticle spacings.

The gradient-POSS laminate exhibited distinct interfacial morphologies among the different interlayers. Relatively smooth, continuous resin regions coexisted with particulate high-contrast regions, and the latter were accompanied by stronger local Si signals. Compared with the uniformly modified 0.5 wt% laminate, large enrichment domains were not continuously observed at every location in the gradient laminate, indicating that the Si-containing phase was concentrated in selected interlayers while the low-content regions retained a relatively continuous resin phase. This observation is consistent with the region-specific application of the 0, 0.3, and 0.5 wt% resin systems. Thus, increasing the nominal POSS content in the uniformly modified laminates produced progressively more pronounced particle enrichment, whereas the gradient laminate exhibited stronger spatial heterogeneity, as expected from its layer-dependent formulation. Large-area EDS mapping showed no obvious Si gradient in the uniformly modified groups, whereas the gradient laminate exhibited a higher Si signal on the nominal high-POSS side and a lower signal toward the POSS-free side, with three broad zones remaining spatially distinguishable. These observations support the presence of a spatially distinguishable stepwise POSS distribution after processing. Because the EDS mapping is semi-quantitative, the exact concentration profile should be interpreted cautiously, and some local redistribution during impregnation and curing may have occurred.

**Figure 4:**
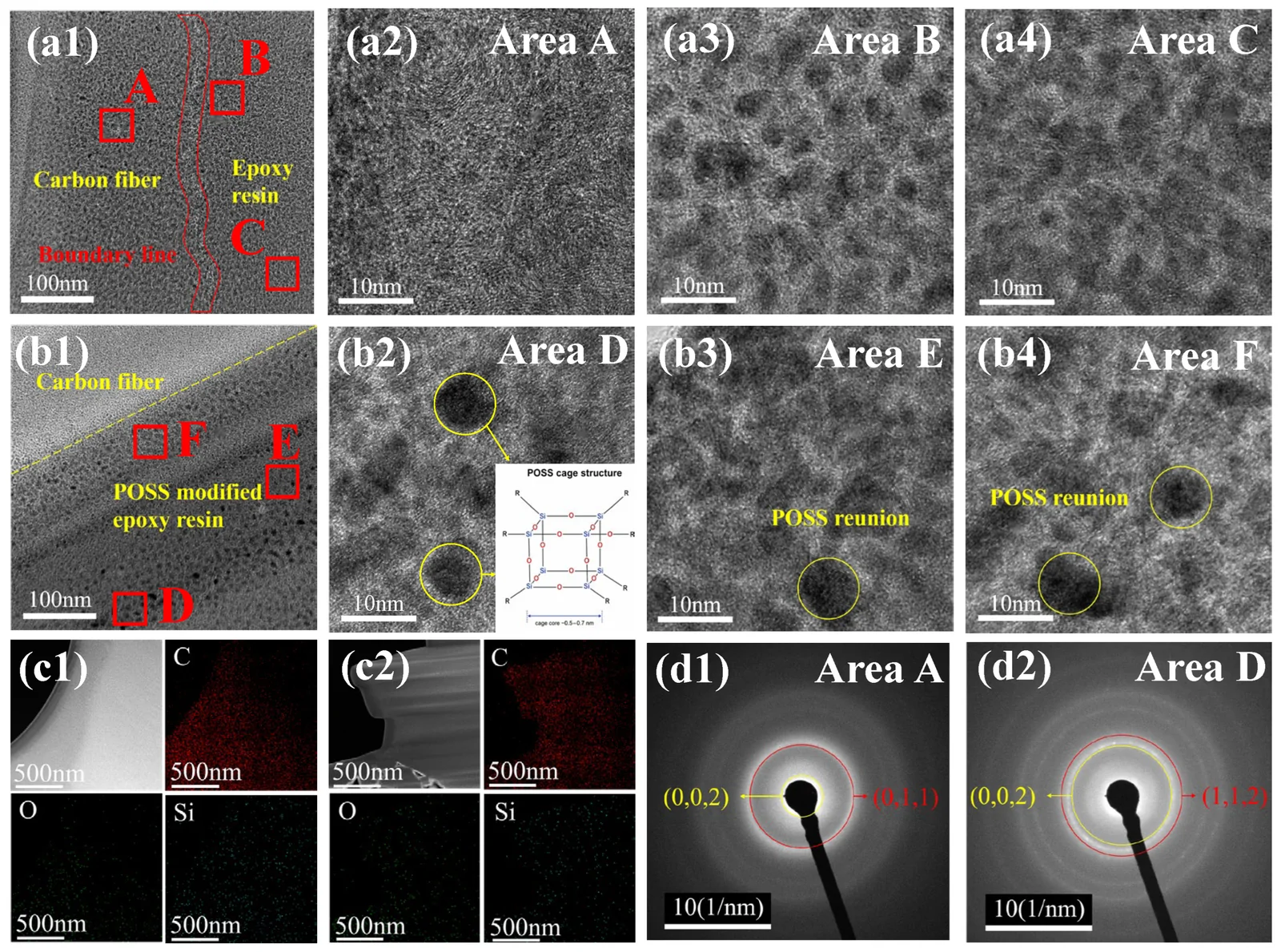
TEM images and EDX elemental maps of two CFRP laminates: (**a1**–**a4**) 0 wt% POSS, (**b1**–**b4**) gradient POSS; (**c1**) EDX elemental maps of the 0 wt% POSS laminate; (**c2**) EDX elemental maps of the gradient-POSS laminate; (**d1**) SAED pattern of the 0 wt% POSS laminate; (**d2**) SAED pattern of the gradient-POSS laminate.

Figure 4 presents TEM observations of nanoscale through-thickness cross-sections from the unmodified and gradient CFRP laminates. Figure 4(a1,b1) show the interfacial regions at lower magnification. In Figure 4(a1), areas A, B, and C were selected from the carbon-fiber region, the interfacial boundary, and the resin region, respectively, and were imaged at high resolution (Figure 4(a2–a4)). In Figure 4(b1), areas D, E, and F were selected from the interfacial boundary and two regions of the POSS-modified resin containing different local POSS concentrations, and the corresponding high-resolution images are shown in Figure 4(b2–b4). Clear boundaries between the carbon fiber and epoxy matrix were visible in both the unmodified and POSS-modified specimens. In Figure 4(a1), the resin side exhibited a relatively uniform amorphous contrast, and no regular particulate structures were observed in areas B and C, consistent with the absence of POSS. Graphitic crystallites and grain boundaries were discernible in area A of the carbon fiber. In the POSS-modified resin in Figure 4(b1), numerous approximately circular dark nanoscale domains, ranging from several nanometers to more than 10 nm, were observed, and their number density and spacing varied among the fields of view. The upper region corresponded to the carbon fiber and exhibited relatively uniform turbostratic-carbon contrast, whereas the lower region corresponded to the POSS-modified epoxy matrix and contained abundant dark nanoscale domains. These darker regions were attributed to POSS-rich domains because the Si-O cages produce stronger electron scattering. Similar domains were observed in areas D–F, indicating that the POSS-containing phase was not completely dispersed at the molecular scale but formed local nanoscale enrichment regions. In the EDX maps in Figure 4(c1,c2), the unmodified region exhibited a weak Si signal, whereas the POSS-modified region showed a more pronounced Si distribution that corresponded to the dark nanoscale domains in the bright-field images.

Figure 4(d1,d2) show selected-area electron diffraction (SAED) patterns obtained from cross-sectional specimens of the unmodified and gradient laminates. The carbon fiber in Figure 4(d1) exhibited broadened diffraction rings, indicating a predominantly turbostratic graphitic structure rather than fully crystalline single-crystal graphite. The inner spacing of d ≈ 0.335 nm corresponds to the (002) interlayer spacing of graphitic carbon; however, this ring overlaps with the central strong-scattering and beam-stop region and is therefore not clearly resolved. The prominent ring at d ≈ 0.20–0.21 nm can be assigned to the graphitic (100)/(101) family. For reference, standard graphite has d002 ≈ 3.355 Å, d100 ≈ 2.132 Å, and d101 ≈ 2.032 Å. The POSS-containing region in Figure 4(d2) exhibited sharper and more numerous diffraction rings, indicating greater local structural ordering. Compared with the carbon-fiber pattern, the rings from the POSS-rich region were sharper and more clearly resolved, suggesting a higher degree of local crystallinity in the examined region.

### 3.2. Mode I Interlaminar Fracture Toughness and Compression-After-Impact Performance

**Figure 5:**
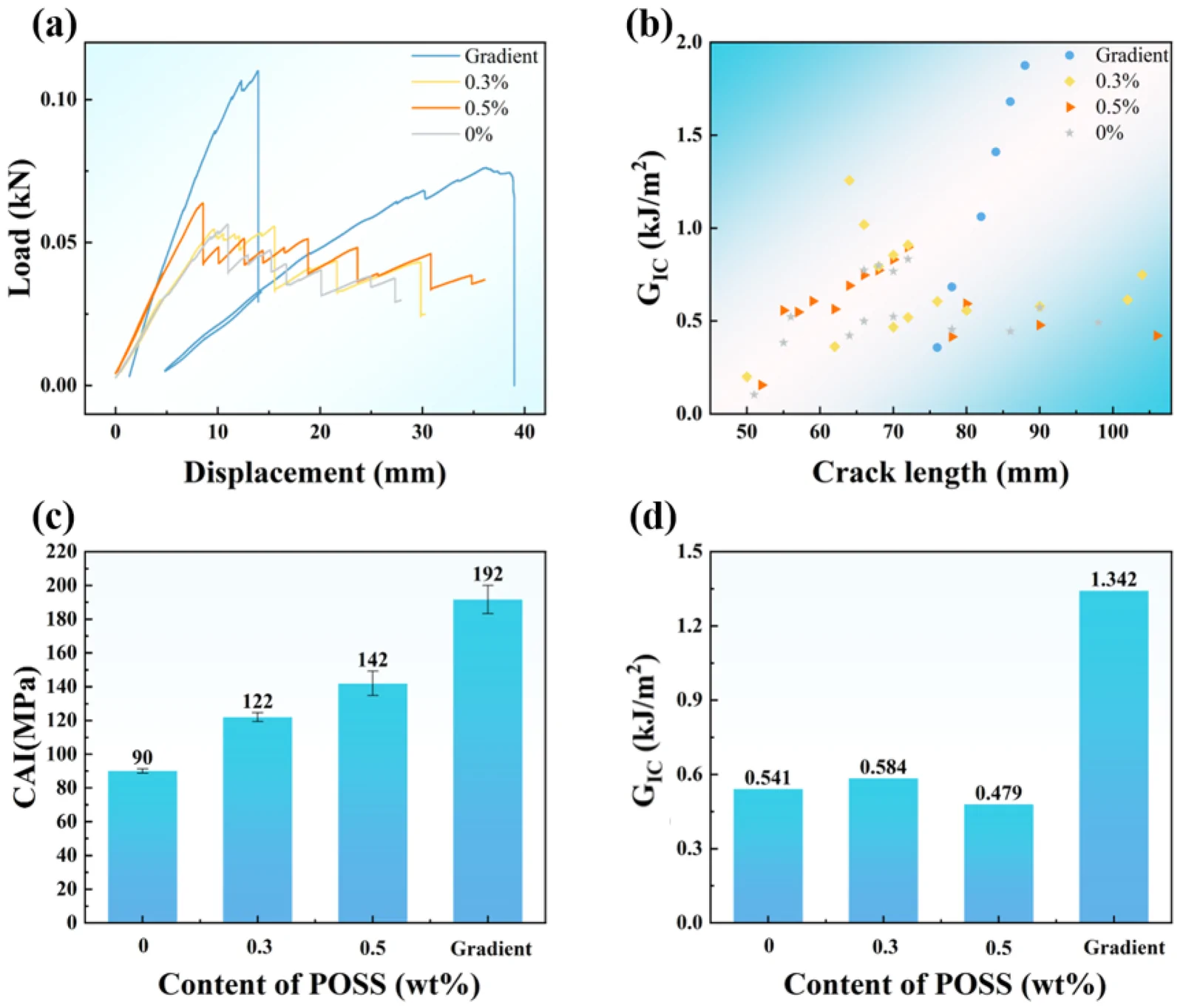
Mode I interlaminar fracture toughness and compression-after-impact performance of CFRP laminates with different POSS contents: (**a**) load–opening-displacement curves; (**b**) R-curves; (**c**) CAI strengths (mean ± SD, *n* = 3); (**d**) GIC values (*n* = 1).

Most measured mechanical properties improved after POSS incorporation. Figure 5a presents the DCB load-opening-displacement curves of the different laminates. All curves initially increased approximately linearly and subsequently exhibited load drops and serrated fluctuations after crack initiation. The peak loads of the unmodified, 0.3 wt%, and 0.5 wt% laminates were similar, whereas the gradient laminate exhibited the highest peak load and maintained a relatively high load over a larger opening-displacement range. These results indicate that the gradient architecture increased both the load required for crack initiation and the sustained resistance during stable crack propagation.

The R-curves in Figure 5b further illustrate the evolution of crack-growth resistance. The GIC values of the unmodified, 0.3 wt%, and 0.5 wt% laminates were mainly distributed between 0.3 and 0.9 kJ·m^−2^ and fluctuated as the crack advanced. By contrast, the R-curve of the gradient laminate continued to rise during the later stage of crack propagation and reached nearly 1.9 kJ·m^−2^. As shown in Figure 5d, the individual characteristic GIC values (*n* = 1) of the unmodified, 0.3 wt%, 0.5 wt%, and gradient laminates were 0.541, 0.584, 0.479, and 1.342 kJ·m^−2^, respectively. Relative to the unmodified laminate, the measured GIC was 7.9% higher for the 0.3 wt% laminate, 11.5% lower for the 0.5 wt% laminate, and approximately 148% higher for the gradient laminate; these are descriptive comparisons of individual DCB measurements. Figure 5c shows the mean CAI strengths with standard-deviation error bars (*n* = 3) for the four groups, which were 90, 122, 142, and 192 MPa, respectively. Relative to the mean unmodified laminate, the 0.3 wt%, 0.5 wt%, and gradient laminates showed descriptive increases of 35.6%, 57.8%, and 113.3%, respectively. Unlike GIC, the CAI strength of the uniformly modified laminates increased continuously with POSS content, indicating that the two metrics are governed by different damage processes. DCB testing primarily characterizes crack initiation and stable growth at a predefined crack front, whereas CAI performance is jointly controlled by the impact-damage area, delamination location, and compressive instability. The gradient laminate achieved the highest values in both tests, indicating that its beneficial effect persisted throughout crack propagation and helped preserve effective load paths after impact.

These results demonstrate that Mode I crack-growth resistance and post-impact compressive load-bearing capacity respond differently to the laminate architecture. Compared with both uniformly modified laminates, the gradient-POSS laminate increased the resistance to crack initiation and propagation while maintaining the highest compressive load-bearing capacity after impact. Because only one DCB specimen was tested for each laminate configuration (*n* = 1), the GIC values reported here represent individual experimental observations. Accordingly, the differences among laminate configurations are descriptive only and should not be interpreted as statistically significant. The CAI values are reported as the mean ± standard deviation of three specimens per configuration (*n* = 3), but the limited sample size does not justify strong claims of statistical significance. Initial tensile/flexural modulus was not measured, so the current data cannot determine whether the gradient changed initial stiffness; the CAI increase refers only to residual compressive strength after impact. The selected gradient was best within the tested set, while broader optimization remains to be undertaken in future work.

### 3.3. Toughening and Damage-Control Mechanisms of Gradient POSS

**Figure 6:**
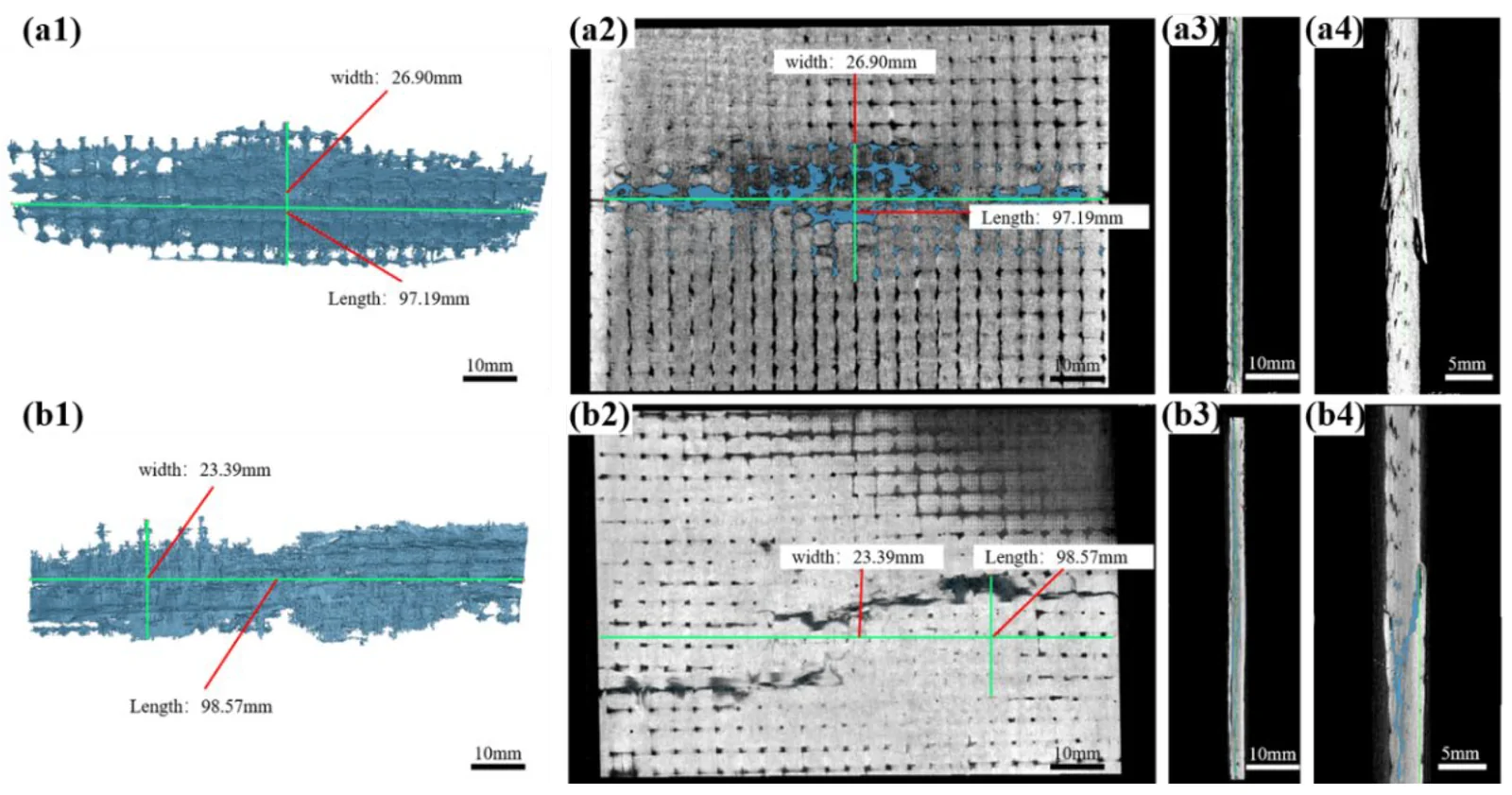
Comparison of internal damage after impact in the 0 wt% POSS and gradient-POSS laminates: (**a1**–**a4**) 0 wt% POSS laminate; and (**b1**–**b4**) gradient-POSS laminate.

The residual specimens after CAI testing were examined by micro-CT from multiple orientations, and the extracted crack morphologies and damage sections are shown in Figure 6. Panels a1 and b1 show the extracted three-dimensional crack morphologies; a2 and b2 show the front-view scans; a3 and b3 show the crack sections along the long-edge direction; and a4 and b4 show the crack sections along the short-edge direction. The damaged region in the unmodified laminate was approximately 97.19 mm long and 26.90 mm wide, whereas that in the gradient laminate was approximately 98.57 mm long and 23.90 mm wide. Damage in both groups extended primarily along the specimen length, but the transverse damage width was smaller in the gradient laminate. The transverse damage width was therefore reduced by approximately 11.2%. The unmodified group showed more continuous internal damage, whereas the gradient group showed a more irregular, segmented profile with local crack deviations. An additional 0.5 wt% scan gave a damage length/width of 99.26/17.61 mm; because equivalent volumetric segmentation was unavailable for all groups, damage volume was not compared quantitatively.

Collectively, the SEM/EDS, TEM, DCB, CAI, and micro-CT results indicate that the role of the gradient POSS distribution can be interpreted at three interrelated levels: interfacial-structure regulation, crack-path reconstruction, and through-thickness functional partitioning. SEM/EDS showed that POSS incorporation increased fracture-surface roughness and produced local Si-enriched regions. TEM further revealed POSS-rich domains ranging from several nanometers to more than 10 nm within the modified resin, while the matrix remained predominantly amorphous. Thus, POSS was not completely dissolved at the molecular scale but existed as a combination of nanoscale domains and local agglomerates. Moderate dispersion can increase interfacial topography and crack-path tortuosity, whereas larger enrichment domains may act as local stress concentrators. Similar competition between dispersion-induced toughening and agglomeration-induced weakening has been reported for nanomodified interlaminar composite systems [15,16,17,47,48].

The uniformly modified laminates illustrate this competition. The slight increase in GIC for the 0.3 wt% POSS laminate indicates that dispersed nanoscale domains at a relatively low concentration provided some resistance to opening-mode crack growth. Although the CAI strength continued to increase for the 0.5 wt% laminate, its GIC decreased. In combination with the more pronounced enrichment domains observed by SEM, this result suggests that reduced local matrix continuity weakened the stable crack-growth resistance ahead of the predefined crack front. This interpretation is a mechanism-based inference from the present morphological and mechanical results and should be further verified through quantitative fractography or in situ crack observation. Within the tested configurations, the gradient laminate achieved the highest measured GIC and CAI values, indicating that spatial distribution can alter the balance between Mode I fracture resistance and post-impact compressive performance. The high-POSS layers show more pronounced interfacial topography and may provide greater local resistance to crack advance; the intermediate-content region forms a transition, whereas the unmodified regions retain a relatively continuous resin phase. The observed crack segmentation is therefore consistent with a crack encountering spatially varying interfacial resistance rather than propagating on a single continuous plane. Such a morphology may increase crack-path tortuosity and local interfacial energy dissipation; however, these processes were not quantified directly. Furtado et al. [47] showed that crack bridging and a nonlinear fracture-process zone can substantially increase the apparent fracture resistance of thin-ply CFRP laminates with nanoscale interlaminar reinforcement. Related studies on CNT-based interlaminar toughening likewise demonstrate that the distribution, wetting, and continuity of the reinforcing phase determine whether it acts as a toughening element or a defect source [15,16,17,47,48].

**Figure 7:**
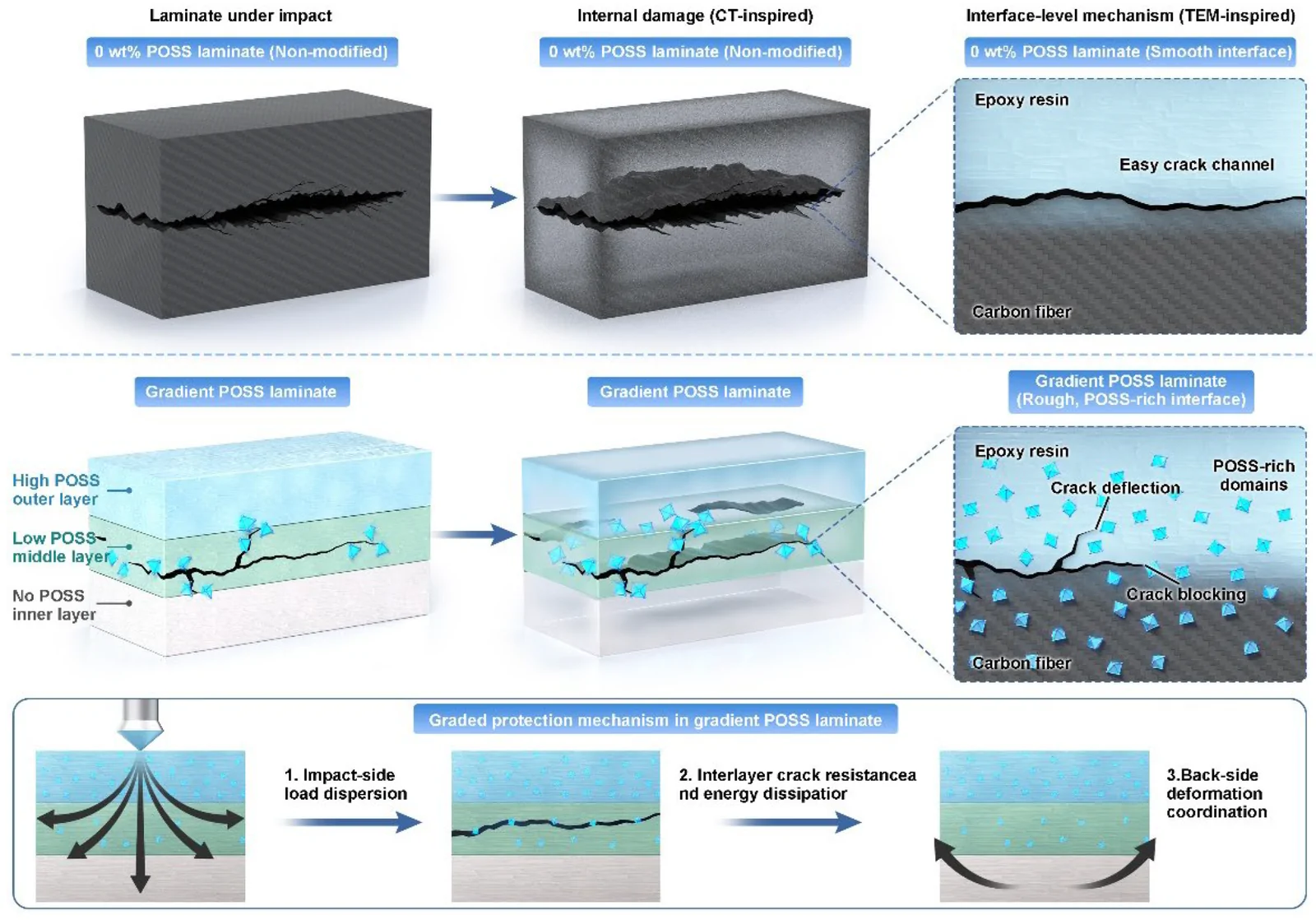
Schematic of the impact-damage-control mechanism in the gradient-POSS laminate.

The impact-damage-control mechanism of the gradient-POSS laminate is illustrated in Figure 7. POSS-rich regions located near crack paths may locally impede crack advancement. Compared with the unmodified laminate, the regions containing different POSS concentrations perform distinct functions within the gradient architecture. The impact-side region with the highest POSS content exhibits more pronounced particle enrichment and interfacial topography, which may help redistribute local contact-induced stresses and restrict lateral damage growth near the impact point. The intermediate region with a lower POSS content provides a transition between interfacial reinforcement and matrix continuity, which may facilitate crack deflection, branching, and frictional energy dissipation. The POSS-free back-side region retains a more continuous resin phase and greater deformation compatibility, allowing it to accommodate tensile deformation generated by impact-induced bending. The nominal stepwise POSS formulation creates spatially varying interfacial states through the thickness, which may contribute to differences in local interlayer resistance and thereby hinder the formation of a single continuous damage plane. The observed segmented crack morphology is consistent with repeated crack deflection and distribution among multiple interlayers, suggesting a graded protection mechanism involving impact-side load redistribution, interlayer crack resistance and energy dissipation, and back-side deformation accommodation [4,25,26,27,28,29,30,31]. This interpretation is consistent with the reduced damage width and lower crack continuity observed for the gradient laminate in Figure 6 and with its simultaneous increases in GIC and CAI strength.

The smaller damage width and reduced crack continuity revealed by micro-CT provide macroscopic evidence for this mechanism. After low-velocity impact, extensive continuous delamination is prone to local buckling under compression and can trigger further damage growth [42,43]. The gradient architecture did not completely eliminate cracking; rather, it restricted lateral crack growth and continuous penetration, thereby preserving a greater proportion of the residual load paths. Its advantage therefore arises not from a single hardening or toughening effect, but from the synergy among interfacial strength, matrix continuity, and crack-growth resistance at different through-thickness locations.

## 4. Conclusions

This study developed a pearl-inspired gradient CFRP laminate designed with a nominal stepwise POSS formulation through the thickness. SEM/EDS, TEM, DCB, CAI, and micro-CT were employed to evaluate its microstructure, mechanical properties, and damage evolution. Compared with the uniformly modified and unmodified laminates, the gradient-POSS laminate showed a higher measured GIC and a higher mean CAI strength in the present tests. These results provide an experimental basis for through-thickness interfacial design and impact-damage control in CFRP laminates. The main conclusions are as follows.

SEM/EDS showed that POSS incorporation increased cross-sectional roughness and enhanced the Si signal near particulate high-contrast regions. The gradient laminate retained both continuous resin-rich regions and POSS-containing regions in different interlayers, producing a heterogeneous morphology consistent with the region-specific lay-up. TEM revealed dark nanoscale domains ranging in size from several nanometers to more than 10 nm in the modified resin, and EDX mapping confirmed their correspondence with Si-enriched regions. SAED indicated that the carbon fibers had a predominantly turbostratic graphitic structure, whereas the epoxy matrix remained largely amorphous; clearer diffraction rings in local regions of the gradient specimen were consistent with the presence of locally ordered POSS-containing domains. In the present tests, the gradient laminate showed the highest measured GIC (1.342 kJ·m^−2^; *n* = 1), and the highest mean CAI strength (192 MPa; *n* = 3), corresponding to descriptive increases of approximately 148% and 113.3%, respectively, relative to the unmodified laminate. Micro-CT showed a reduction in damage width and crack continuity in the gradient laminate. The combined results are consistent with a beneficial combination of interfacial roughening, altered crack paths, and matrix continuity associated with the through-thickness POSS distribution, which may contribute to the higher measured fracture resistance and residual post-impact load-bearing capacity. These findings provide an experimental basis for the through-thickness interfacial design of high-damage-tolerance CFRP laminates for aerospace, rail transportation, and protective structures, and offer guidance for structurally tailored toughening strategies. Because the DCB results are based on one specimen per laminate configuration (*n* = 1), the reported GIC differences are individual experimental observations and no statistically significant differences among configurations are claimed. The CAI results are based on the mean ± standard deviation of *n* = 3 specimens and should still be interpreted cautiously. Initial stiffness was not measured and should be evaluated in future modulus tests.

## Data Availability

The original contributions presented in this study are included in the article/Supplementary Materials. Further inquiries can be directed to the corresponding author.

## Supplementary Materials

The following supporting information can be downloaded at: https://www.mdpi.com/article/10.3390/polym18182199/s1, Figure S1: Large-area SEM images and EDS elemental maps of C, O, and Si across the through-thickness sections of CFRP laminates: (a) 0 wt% POSS; (b) 0.3 wt% POSS; (c) 0.5 wt% POSS; and (d) gradient-POSS; Figure S2: Micro-CT scan of the 0.5 wt% POSS-modified laminate; Figure S3: CAI values for all CFRP laminates; Figure S4: DSC curves of epoxy castings with different POSS contents; Figure S5: FTIR spectra of POSS, neat epoxy resin, and POSS-modified epoxy resin; Figure S6: DMA tan δ curves of epoxy resins with different POSS contents.
