# Supplementary material for: Performance of Multi-Gradient Biomimetic POSS-Toughened CFRP Laminates with a Pearl-Inspired Hard-Soft Interfacial Architecture
Source: Polymers (Basel). 2026 Sep 9;18(18):2199. doi: 10.3390/polym18182199 (PMC13610891; doi:10.3390/polym18182199)
Supplement: Supplementary file 1 [file polymers-18-02199-s001.zip › polymers-4524177-supplementary.pdf]

Supplementary Materials

Performance of Multi-Gradient POSS-Toughened CFRP Laminates with a Pearl-Inspired Gradient Interfacial Architecture

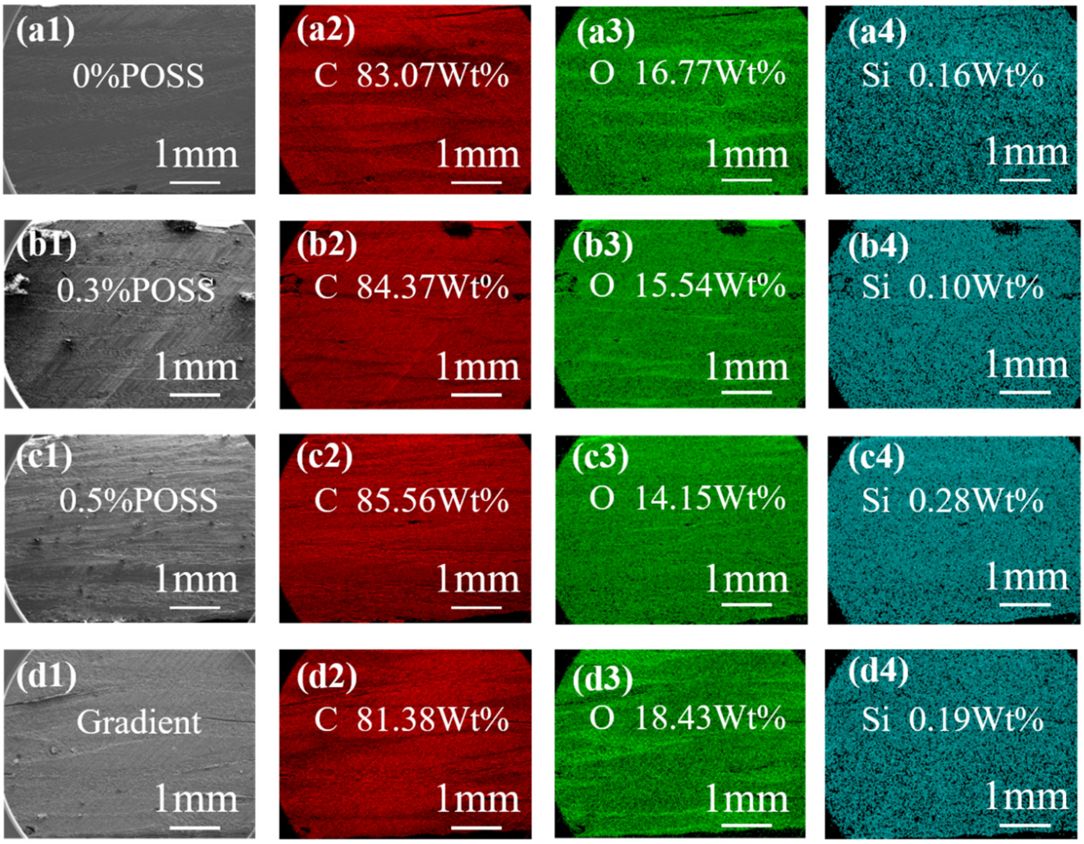

Figure S1. Large-area SEM images and EDS elemental maps of C, O, and Si across the through-thickness sections of CFRP laminates: (a) 0 wt% POSS; (b) 0.3 wt% POSS; (c) 0.5 wt% POSS; and (d) gradient-POSS.

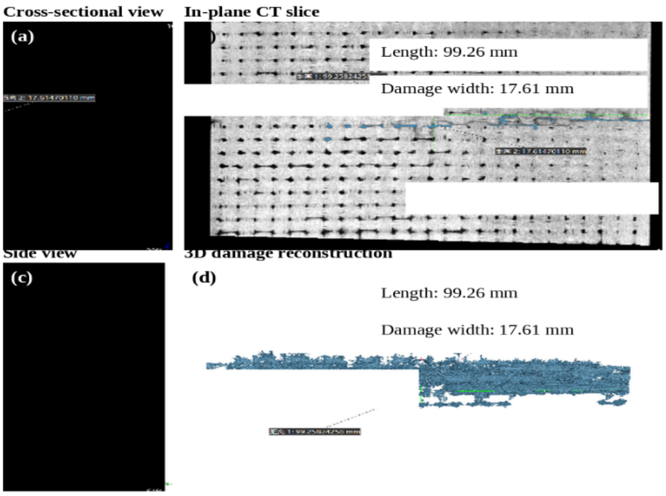

Figure S2. Micro-CT scan of the 0.5 wt% POSS-modified laminate.

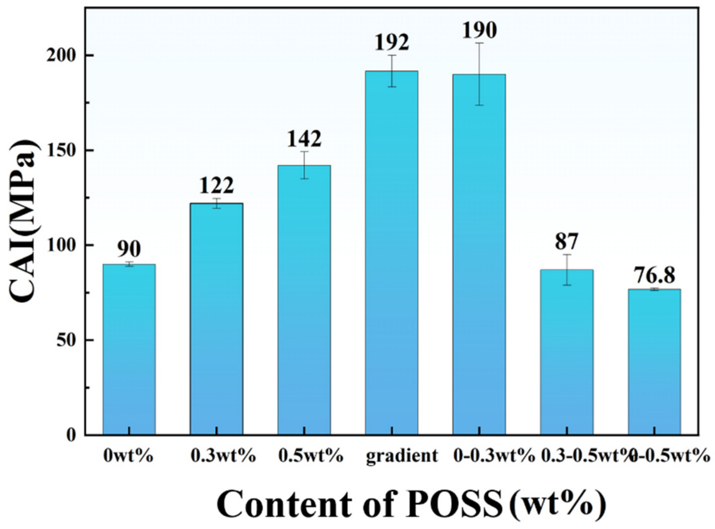

Figure S3. CAI values for all CFRP laminates.

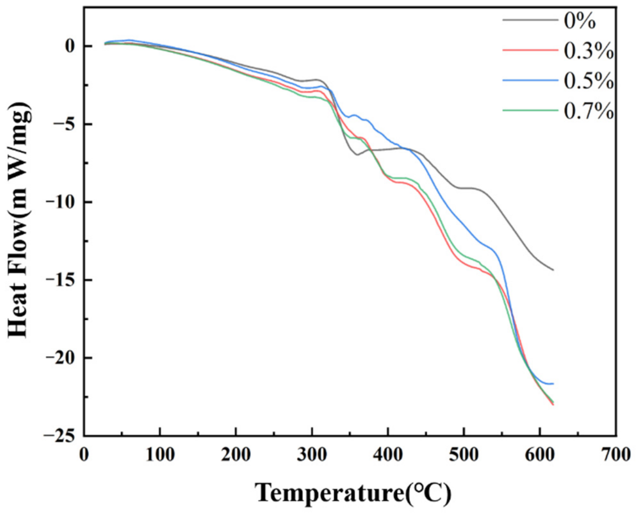

Figure S4. DSC curves of epoxy castings with different POSS contents.

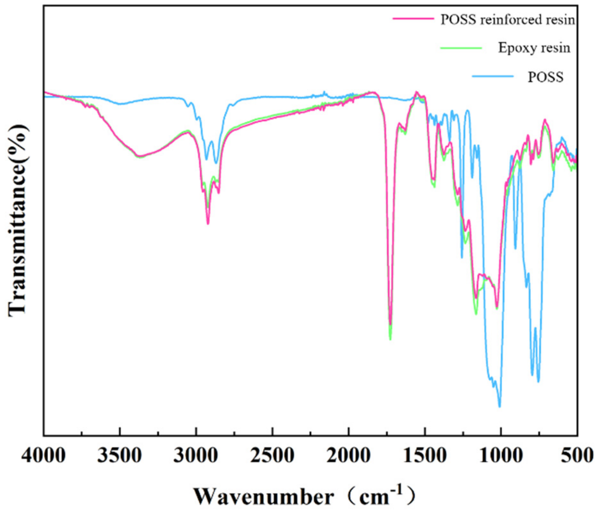

Figure S5. FTIR spectra of POSS, neat epoxy resin, and POSS-modified epoxy resin.

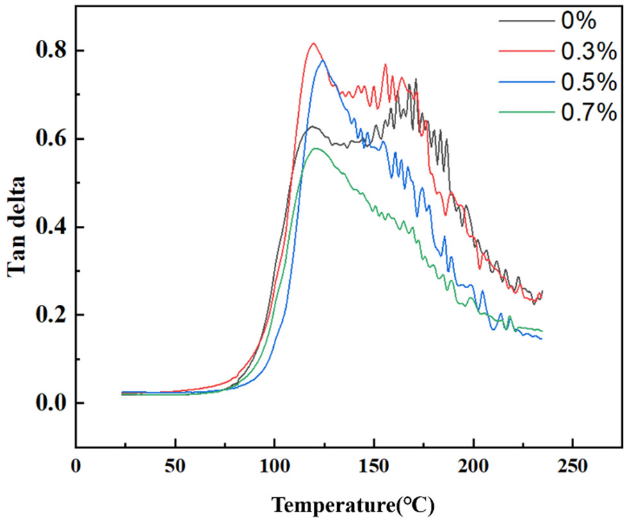

Figure S6. DMA tan δ curves of epoxy resins with different POSS contents.
